# Romantic Psychology: A Strange Story and Elsie Venner

**Published:** 1862-07

**Authors:** 


### Art. V.—ROMANTIC PSYCHOLOGY: A STRANGE STORY AND ELSIE VENNER.

Time was when philosophy was romantic; now romance has become philosophic. Subject to a similar law of mental development, both the philosopher and the writer of fiction, whether poet or novelist, have sought, each in his own sphere, to curb within straiter limits the mobile imagination. Reason and imagination are so closely linked together that any affection of the one reacts upon the other. Bacon has quaintly described the imagination as an agent or "nuncius" between the judicial (knowing and reasoning—cognitive) and ministerial (willing and desiring—conative)

provinces of the mind of man. "For," he says, "sense sendeth over to imagination before reason have judged, and reason sendeth over to imagination before the decree can be acted; for imagination ever precedeth voluntary motion. . . . . Neither is the imagination simply and only a messenger, but is invested with, or at least usurpeth, no small authority in itself besides the duty of the message. For it was well said by Aristotle, 'That the mind hath over the body that commandment which a magistrate hath over a free citizen, who may come also to rule in his turn.'"* But the action of the messenger is sometimes sluggish and feeble, oft-times too vivacious or perverted. "Every one is jostled, but some are quite overthrown by the imagination," says Montaigne;† and Hume has finely observed that "nothing is more dangerous to reason than the flights of imagination, and nothing has been the occasion of more mistakes among philosophers. Men of bright fancy may, in this respect, be compared to those angels whom the Scriptures represent as covering their eyes with their wings."‡

Taught by the bitter experience of errors and follies, so deeply graven in its history, philosophy has long sought to restrain the imagination within more contracted bounds—nay, it has tended even (very naturally must be confessed) to exclude the latter altogether from its domains. Yet it is not to be overlooked that, whatever the danger which may arise of usurpation over the understanding and reason, the promptings of the imagination are a necessary condition of philosophy as well as of poetry and romance. "A vigorous power of representation," says Sir William Hamilton, "is as indispensable a condition of success in the abstract sciences as in the poetical and plastic arts; and it may, accordingly, be reasonably doubted whether Aristotle or Homer were possessed of the more powerful imagination."§ He adds, quoting from Ancillon, "We may, indeed, affirm that there are as many different kinds of imagination as there are different kinds of intellectual activity. There is the imagination of abstraction, which represents to us certain phases of an object to the exclusion of others, and, at the same time, the sign by which the phases are united; the imagination of wit, which represents differences and contrasts, and the resemblances by which these are combined; the imagination of judgment, which represents the various qualities of an object and binds them together under the relations of substance, of attribute, of mode; the imagination of reason, which represents a principle in connexion with its consequences, the effect in de-

---

* *Advancement of Learning*, bk. ii. † *Essays*, ch. xx.

‡ *Treatise on Human Nature*, bk. i. pt. iv. § 7, cited by Sir William Hamilton.

§ *Lectures on Metaphysics*, vol. ii. p. 265.

pendence on its cause; the imagination of feeling, which represents the accessory images kindred to some particular sentiment, and which thereby confer on it greater compass, depth, and intensity; the imagination of volition, which represents all the circumstances which concur to persuade or dissuade from a certain act of will; the imagination of the passions, which, according to the nature of the affection, represents all that is homogeneous or analogous; finally, the imagination of the poet, which represents whatever is new, beautiful, or sublime—whatever, in a word, it is determined to represent by any interest or art."*

Elsewhere Sir William Hamilton writes: "The vigour and perfection of this faculty (imagination) is seen not so much in the representation of individual objects and fragmentary sciences, as in the representation of systems. In the better ages of antiquity, the perfection—the beauty, of all works of taste, whether in poetry, eloquence, sculpture, painting, or music, was principally estimated from the symmetry or proportion of all the parts to each other, and to the whole which they together constituted; and it was only in subservience to this general harmony that the beauty of the several parts was appreciated. In the criticism of modern times, on the contrary, the reverse is true, and we are disposed to look more to the obtrusive qualities of details than to the keeping and unison of a whole. . . . . The reason of this difference in taste seems to be, what at first sight may seem the reverse, that in antiquity, not the reason, but the imagination, was the most vigorous; that the imagination was able to represent simultaneously a more comprehensive system; and thus the several parts being regarded and valued only as conducive to the general result—these parts never obtained that individual importance which would have fallen to them had they been only created, and only considered for themselves. Now this power of representing to the mind a complex system in all its bearings is not less requisite to the philosopher than to the poet, though the representation be different in kind; and the nature of the philosophic representations, as not concrete and palpable like the poetical, supposes a more arduous operation, and therefore, even a more vigorous faculty. But imagination, in the one case and in the other, requires in proportion to its own power a powerful intellect; for imagination is not poetry, nor philosophy, but only the condition of the one and the other."†

The character and functions of poesy have been so admirably expressed by Bacon, and in a manner so pertinent to our present purpose, that it would be folly to substitute other words for his.

---

* *Essais Philosophiques,* vol. ii. p. 151.
† *Lectures on Logic,* vol. ii. pp. 131–32.

There is a peculiar fitness, moreover, in quoting Bacon's observations on this subject, since to him chiefly is to be assigned that marvellous restoration, or, as he phrases it, instauration, of philosophy, an indirect, but not unimportant, and certainly most interesting effect of which is to be noted in the philosophical romances of recent times. "POESY," he says, "is a part of learning in measure of words for the most part restrained, but in all other points extremely licensed, and doth truly refer to the imagination, which, being not tied to the laws of matter, may at pleasure join that which nature hath severed, and sever that which nature hath joined; and so make unlawful matches and divorces of things." Of the matter of poesy, he says: that it is "one of the principal portions of learning, and is nothing else but feigned history, which may be styled as well in prose as in verse." He then proceeds: "The use of this feigned history hath been to give some shadow of satisfaction to the mind of man in those points wherein the nature of things doth deny it, the world being in proportion inferior to the soul; by reason whereof there is, agreeable to the spirit of man, a more ample greatness, a more exact goodness, and a more absolute variety, than can be found in the nature of things. Therefore, because the acts or events of true history have not that magnitude which satisfieth the mind of man, poesy feigneth acts and events greater and more heroical; because true history propoundeth the successes and issues of actions not so agreeable to the merits of virtue and vice, therefore poesy feigns them more just in retribution, and more according to revealed providence: because true history representeth actions and events more ordinary, and less interchanged, therefore poesy endueth them with more rareness, and more unexpected and alternative variations: so that it appeareth that poesy serveth and conferreth to magnanimity, morality, and to delectation. And therefore it was ever thought to have some participation of divineness, because it doth raise and erect the mind by submitting the shews of things to the desires of the mind; whereas reason doth buckle and bow the mind unto the nature of things. And we see, that by these insinuations and congruities with man's nature and pleasure, joined also with the agreement and consort it hath with music, it hath had access and estimation in rude times and barbarous regions, when other learning stood excluded."*

Now, prior to Bacon's time, the imagination usurped almost as great sway over philosophy as it most legitimately exercised over poesy, and he sought to "add lead and ballast to the understanding, to prevent its jumping or flying,"† which had not up to that time been done. "True philosophy," he taught, "is that

* *Advanct. of Learning*, bk. ii. † *Nov. Org.* aph. 104.

which is the faithful echo of the voice of the world, which is written in some sort under the dictation of things, which adds nothing of itself, which is only the rebound, the reflection of reality." He, therefore, maintaining the great, the fundamental truth in all sound reasoning, that the "understanding left to itself possesses but little power,"* set up a light† for its guidance, and ballasted it with a justly-regulated and digested method of procedure. He thus enabled the reason more closely to "buckle and bow the mind unto the nature of things," by showing the true path of experience, and by restraining the understanding, prompted by a restless imagination, from "jumping and flying from particulars to remote and more general axioms (such as are termed the principles of arts and things), and thus prove and make out their intermediate axioms according to the supposed unshaken truth of the former."‡

The mighty influence exercised by Bacon's teaching in emancipating the understanding from those trammels which beset it, and promoting the just exercise of the reason—most conspicuously manifested in the natural sciences—has not been restricted solely to the sphere of philosophy proper. For it has extended, and with equal benefit, to all liberal education whatever, and with the same result (so far as we are at present concerned) in both philosophy and education—the restraint of the imagination within limits more consistent with the nobility both of the reason and understanding. Hence even the writer of fiction in the present day, as well from natural inclination as from the readier acceptation accorded to his labours, seeks to render his imaginative powers subordinate to the true nature of things, and to educe from them a just and legitimate moral, rather than to override truth by fanciful creations. He does not seek to display either "a more ample greatness, a more exact goodness, or a more absolute variety," than humanity gives a warrant for. He does not feign acts and events greater and more heroical than those which true history recounts. He does not make virtue more virtuous, nor vice more vicious, neither does he accord nobler rewards to the one nor a juster retribution to the other, than the experience of ordinary life teaches. But his art not the less, nay, even more truly, "serveth and conferreth to magnanimity, morality, and to delectation." For his genius pierceth the inmost recesses and wondrous workings of the soul; and while charming the imagination and enthralling the attention with a fictitious story and characters, he by means of these lays more fully bare and scarifies more deeply the follies, vices, and weaknesses, as well as brings more clearly to light and ennobles the

---

* *Nov. Org.* aph. 2. † Ibid. 82. ‡ Ibid. 104.

virtues of the individual and the many. He makes the imagination, vitalized by genius, subservient to Reason and the Truth, and while ministering to the latter, he adds greater lustre to the former. Whether he derives the materials upon which to exercise his powers from history, domestic or social life; whether he writes of love or hate, of grief or joy, of crime or innocence, of peace or war, of religion, science, or philosophy, of the present, the past, or the future, whatever his brilliancy of fancy, his fertility of invention, or his happiness of expression, he alone will be accepted as a master of his craft whose ideal portraiture is conceived and executed in the spirit of truth.

> "Poets are all who love, who feel great truths,
> And tell them;"

has sung one of the rarest spirits of modern times—one who in a fanciful web has woven constellations of burning truths. It is in the recognition of this truth in its entirety, not as confined to abstract and transcendental truths, but as including those concrete truths with which we have to deal in every-day life, that we entertain the hope that the poet and his congeners, the novelist and dramatist, will not fail to play their part, holily and manfully, in the great and persistent struggle to drive truth and goodness home to the fiery marrow of the world—a struggle which at no time ever called so loudly for the strenuous exercise of every power of the intellect to carry on its widening ascendancy as now. And the hope is not a vain one, for we lack neither poets, nor novelists, nor dramatists who, deeply tinctured themselves by the stern, sober spirit of the times, react upon and intensify that spirit—taught and teaching. "'Tis impossible," exclaims one of the chiefest of these, one, alas! whose words now come to us hallowed by death—

> "'Tis impossible
> To get at men excepting through their souls,
> However open their carnivorous jaws;
> And poets get directer at the soul
> Than any of your economists: for which,
> You must not overlook the poet's work
> When scheming for the world's necessities.
> The soul's the way."*

In no respect is a conscientious regard to the true spirit of his art more imperatively demanded of the poet and the novelist than when he deals with questions peculiar to science or philosophy, in which the multitude are at the mercy of the writer who most

* *Aurora Leigh*, bk. viii.

fully enchains the imagination and attention. There was a time when poets "forged gods—uttered, made them pass." But now we look to him and his congeners to bedeck truth—to make its mighty influence still mightier—and not to create lies. Hence a higher interest than that which arises out of a pleasant exercise of the imagination is attached to those writings which appeal to the reason through the fancy, and under the guise of a fiction seek to inculcate a great philosophical or scientific truth. Two works of this class have recently issued from the press, "A Strange Story," by Sir E. Bulwer Lytton, and "Elsie Venner," by Oliver Wendell Holmes, and have created unusual interest. Both works are remarkable illustrations of the tendency of writers of fiction, in modern times, and particularly in our own days, to infuse into their works a loftier purpose than that of simply gratifying the imagination.

It is fortunate that the authors referred to have themselves given the clue to their own works. This was especially needed in regard to "A Strange Story," for the conflict of opinion which arose as to its object while it was being doled out in the pages of a serial, was bewildering. The completed work is, however (most happily for the reader), preceded by a preface which is not the least remarkable portion of the book, and which would seem to have been written to meet the many doubts which beset the public during its publication.

"Elsie Venner" originally appeared in successive parts of the *Atlantic Monthly*, under perhaps the more fitting title of "The Professor's Story." It also, in its finished state, is preceded by a preface, from which we learn that—

"In calling this narrative a 'romance,' the author wishes to make sure of being indulged in the common privileges of the poetic licence. Through all the disguise of fiction a grave scientific doctrine may be detected lying beneath some of the delineations of character. He has used this doctrine as a part of the machinery of his story, without pledging his absolute belief as to the extent to which it is asserted or implied. It was adopted as a convenient medium of truth rather than as an accepted scientific conclusion."

The author of "A Strange Story" begins his preface with a brief notice of the perfected philosophy of Maine de Biran, who is described by Victor Cousin as the most original of the many illustrious thinkers whom the schools of France have contributed to the intellectual philosophy of our age.

"In the successive developments of his own mind," Sir E. B. Lytton says, "Maine de Biran may, indeed, be said to represent the change that has been silently at work throughout the general mind of

Europe since the close of the last century. He begins his career of philosopher with blind faith in Condillac and Materialism. As an intellect, severely conscientious in the pursuit of truth, expands amidst the perplexities it revolves, phenomena which cannot be accounted for by Condillac's sensuous theories open to his eye. To the first rudimentary life of man, the animal life, 'characterized by impressions, appetites, movements, organic in their origin, and ruled by the Law of Necessity :'* he is compelled to add 'the second, or human life, from which Free-will and Self-consciousness emerge.' He thus arrives at the union of mind and matter; but still a something is wanted, some key to the marvels which neither of these conditions of vital being suffice to explain. And at last the grand self-completing Thinker arrives at the Third Life of Man in Man's Soul. 'There are not,' says this philosopher, towards the close of his last and loftier work—'there are not only two principles opposed to each other in Man, there are three; for there are, in him, three lives and three orders of faculties. Though all should be in accord and in harmony between the sensitive and the active faculties which constitute Man, there would still be a nature superior—a third life, which would make felt (*ferait sentir*) the truth that there is another happiness, another wisdom, another perfection, at once above the greatest human happiness, above the highest wisdom, or intellectual and moral perfection of which the human being is susceptible.' "†

Sir E. B. Lytton then proceeds :—

"Now, as Philosophy and Romance both take their origin in the Principle of Wonder, so in the Strange Story submitted to the Public, it will be seen that Romance, through the freest exercise of its wildest vagaries, conducts its bewildered hero towards the same goal to which Philosophy leads its luminous student, through far grander portents of Nature, far higher visions of Supernatural Power, than Fable can yield to Fancy. That goal is defined in these noble words, 'The relations (*rapports*) which exist between the elements and the products of the three lives of Man are the subject of meditation, the fairest and finest, but also the most difficult. The Stoic Philosophy shows us all which can be most elevated in active life, but it makes abstraction of the animal nature, and absolutely fails to recognise all which belongs to the life of the spirit. Its practical morality is beyond the forces of humanity. Christianity alone embraces the whole Man. It dissimulates none of the sides of his nature, and avails itself of his miseries and his weakness in order to conduct him to his end, in showing him all the want he has of a succour more exalted.' "‡

In the passages thus quoted, Sir. E. B. Lytton implies one of the objects for which his tale has been written, and he cites them "with a wish to acknowledge one of those priceless obligations which writings, the lightest and most fantastic, often incur to

* *Œuvres inédites de Maine de Biran,* vol. i. See Introduction.

† *Op. cit.* vol. iii. p. 546 (Anthropologie). ‡ *Op. cit.* vol. iii. p. 524.

reasoners the most serious and profound." But, adding a caution, he remarks that "he here constructs a romance which should have, as a romance, some interest for the general reader;" he does not "elaborate a treatise submitted to the logic of sages." He further adds,—"It is only when 'in fairy fiction drest,' that romance gives admission to 'truths severe.'"

Our author next proceeds to illustrate the principle admitted by critics, that "a supernatural machinery is indispensable" to the Epic. The reason of this, he holds, "is that the Epic is the highest and the completest form in which art can express either man or nature, and that without some gleams of the supernatural, man is not man, nor nature, nature." In support of this proposition he quotes certain observations of "the pious and profound Jacobi" (as Sir W. Hamilton terms him). "Is it unreasonable," writes Jacobi, "to confess that we believe in God, but by reason of the Supernatural in Man which alone reveals and proves him to exist? . . . . Man reveals God: for man, by his intelligence, rises above nature: and in virtue of this intelligence is conscious of himself as a power not only independent of, but opposed to nature; and capable of resisting, conquering, and controlling her." Now, if the meaning involved in Jacobi's argument be carefully studied, Sir E. B. Lytton believes that "we shall find deeper reasons than the critics who dictated canons of taste to the last century discovered—why the supernatural is indispensable to the Epic, and why it is allowable to all works of imagination, in which art looks on nature with man's inner sense of a something beyond and above her." He then notes, touching upon that changed and more sober taste of the present age in works of imagination to which we have already referred, that—

"The writer who, whether in verse or prose, would avail himself of such sources of pity or terror as flow from the Marvellous, can only attain his object in proportion as the wonders he narrates are of a kind to excite the curiosity of the age he addresses. In the brains of our time," he continues, "the faculty of *Causation* is very markedly developed. People, now-a-days, do not delight in the Marvellous, according to the old child-like spirit. They say in one breath, 'Very extraordinary,' and in the next breath ask, 'How do you account for it?' If the Author of this work has presumed to borrow from science some elements of interest for Romance, he ventures to hope that no thoughtful reader—and certainly no true son of science—will be disposed to reproach him. In fact, such illustrations from the masters of Thought were essential to the completion of the purpose which pervades the work. That purpose, I trust, will develope itself in proportion as the story approaches the close."

He adds, finally:—

"Of course, according to the most obvious principles of art, the narrator of a fiction must be as thoroughly in earnest as if he were the narrator of facts. One could not tell the most extravagant fairy tale so as to rouse and sustain the attention of the most infantine listener, if the tale were told as if the tale-teller did not believe in it. But when the reader lays down this Strange Story, perhaps he will perceive through all the haze of Romance the outlines of these images suggested to his reason:—Firstly, the image of sensuous, soulless Nature, such as the Materialist had conceived it. Secondly, the image of Intellect, obstinately separating all its inquiries from the belief in the spiritual essence and destiny of man, and incurring all kinds of perplexity and resorting to all kinds of visionary speculation, before it settles at last into the simple faith which unites the philosopher and the infant. And, thirdly, the image of the erring, but pure-thoughted visionary, seeking overmuch on this earth to separate soul from mind, till innocence itself is led astray by a phantom, and reason is lost in the space between earth and the stars. Whether in these pictures there be any truth worth the implying, every reader must judge for himself; and if he doubt or deny that there be any such truth, still in that process of thought which the doubt or denial enforces, he may chance on a truth which it pleases him to discover."

Thus far the preface to the "Strange Story." To this romance we shall first confine our attention. We assume that our readers are familiar with the story as a story. With the tale and its general interest as such we have little to do here. Our task is limited to an examination of the philosophical elements imported into it, as stated by the author, and the psychological considerations arising out of them. It is manifest, then, that the chief interest for us centres in the "image of Intellect" (represented in the character of Dr. Fenwick) at first scorning and contemning all faith and research in the spiritual, but subsequently settling "into the simple faith which unites the philosopher and the infant." We have to learn in what manner, and by what mechanism Dr. Fenwick, beginning his career "with a blind faith in Condillac and Materialism," in progress of time, impelled by the Principle of Wonder, in which "both Philosophy and Romance take their origin," comes, after Maine de Biran, to link the Animal Life to the Human Life, "from which Free-Will and Self-Consciousness emerge," and finally attains to a knowledge of the "Third Life of Man in Man's Soul." We have to learn what was the fashion of that Wonder, what the nature of the "supernatural agency"—the Marvellous—evoking the Principle, which led him to an acquaintance "with the truth that there is another happiness, another wisdom, another perfection, at once above the greatest human happiness, above the highest wisdom, or intellectual and moral perfection of which a human being is susceptible." We have, in short, to learn by what means Romance, when written by a master in the craft, may, "through the freest exercise of its wildest vagaries," conduct its bewildered hero to the same goal to which philosophy leads its luminous student, to wit, the recognition of all which belongs to the life of the spirit; and of the great practical truth, that "Christianity alone embraces the whole man."

It would almost seem as if Sir E. B. Lytton gave us to understand that his "Strange Story" is a kind of Philosophical Pilgrim's Progress, adapted to certain wants of the present age. Let us hasten, then, to know the Pilgrim and to mark how romance comports itself in the strait path of philosophy.

Our Pilgrim is a physician—one Dr. Fenwick. We are to conceive of him as having studied his art and science where best that art and science may be studied; and as having won "whatever guarantees for future distinction the praise of professors may concede to the ambition of students." He is already an author and authority on one professional subject, to which his aspirations for fame are by no means limited. Accident, while travelling in the Tyrol, throws him in the way of a certain Julius Faber, "a physician of great distinction, contented to reside where he was born, in the provincial city of L——, but whose reputation as a profound and original pathologist was widely spread, and whose writings had formed no unimportant part of his special studies." He has the good hap to succour this gentleman in serious illness; hence a warm attachment on the part of the older to the younger physician, "perhaps the more affectionate because he was a childless bachelor, and the nephew who would succeed to his wealth evinced no desire to succeed to the toils by which the wealth had been acquired." Thus, having an heir for the one, he had long looked out for an heir to the other, and now resolved to find that heir in Dr. Fenwick. He induces him to forego an intention to practise his profession in London, and to assist him in the city of L——; and in that place the first scene of the romance is played out. There we find Dr. Fenwick established, a young but successful practitioner. Presently Dr. Faber leaves the field entirely to his young partner, and devotes himself to foreign travel; but when needed, he plays the part of mentor throughout the story.

The mental characteristics of our hero are so aptly limned that we cannot omit one line of the picture. He is the relator of his own story, it will be remembered.

"I know not," he says, "whether a success far beyond that usually attained at the age I had reached served to increase, but it seemed to myself to justify the main characteristic of my moral organization—intellectual pride.

"Though mild and gentle to the sufferers under my care, as a necessary element of professional duty, I was intolerant of contradiction from those who belonged to my calling, or even from those who, in general opinion, opposed my favourite theories.

"I had espoused a school of medical philosophy severely rigid in its inductive logic. My creed was a stern materialism. I had a contempt for the understanding of men who accepted with credulity what they could not explain by reason. My favourite phrase was 'common sense.' At the same time I had no prejudice against bold discovery, and discovery necessitates conjecture, but I dismissed as idle all conjecture that could not be brought to a practical test.

"As in medicine I had been the pupil of Broussais, so in metaphysics I was the disciple of Condillac. I believed with that philosopher that 'all our knowledge we owe to Nature, that in the beginning we can only instruct ourselves through her lessons, and that the whole art of reasoning consists in continuing as she has compelled us to commence.' Keeping natural philosophy apart from the doctrines of revelation, I never assailed the last, but I contended that by the first no accurate reasoner could arrive at the existence of the soul as a third principle of being, equally distinct from mind and body. That by a miracle man might live again, was a question of faith and not of understanding. I left faith to religion, and banished it from philosophy. How define, with a precision to satisfy the logic of philosophy, what was to live again? The body? We know that the body rests in its grave till by the process of decomposition its elemental parts enter into other forms of matter. The mind? but the mind was as clearly the result of the bodily organization as the music of the harpsichord is the result of the instrumental mechanism. The mind shared the decrepitude of the body in extreme old age; in the full vigour of youth a sudden injury to the brain might for ever destroy the intellect of a Plato or a Shakspeare. But the third principle—the soul—the something lodged within the body, which yet was to survive it? Where was that soul hidden out of the ken of the anatomist? When philosophers attempted to define it, were they not compelled to confound its nature and its actions with those of the mind? Could they reduce it to the mere moral sense, varying according to education, circumstances, and physical constitution? But even the moral sense in the most virtuous of men may be swept away by fever. Such at the time I now speak of were the views I held; views certainly not original nor pleasing, but I cherished them with as fond tenacity as if they had been consolatory truths of which I was the first discoverer. I was intolerant to those who maintained opposite doctrines, despised them as irrational, or disliked them as insincere. Certainly, if I had fulfilled the career which my ambition predicted, become the founder of a new school in pathology, and summed up my theories in academical lectures, I should have added another authority, however feeble, to the sects which circumscribe the interest of man to the life that has its close in the grave."

What follows is admirable as the expression of a profound psychological truth.

"Possibly that which I have called my intellectual pride was more nourished than I should have been willing to grant by that self-reliance which an unusual degree of physical power is apt to bestow. Nature had blessed me with the thews of an athlete. Among the hardy youths of the northern Athens I had been pre-eminently distinguished for feats of activity and strength. My mental labours, and the anxiety which is inseparable from the conscientious responsibilities of the medical profession, kept my health below the par of keen enjoyment, but had in no way diminished my rare muscular force. I walked through the crowd with the firm step and lofty crest of the mailed knight of old, who felt himself, in his casement of iron, a match against numbers. Thus the sense of a robust individuality, strong alike in disciplined reason and animal vigour, habituated to aid others, needing no aid for itself, contributed to render me imperious in will and arrogant in opinion. Nor were such defects injurious to me in my profession; on the contrary, aided as they were by a calm manner and a presence not without that kind of dignity which is the livery of self-esteem, they served to impose respect and inspire trust."

Such is our hero, and we have now to follow, step by step, the events which in the end proved influential in subjugating his intellectual pride.

The chief professional rival of Dr. Fenwick, in the city of L——, was a Dr. Lloyd, "a benevolent, fervid man, not without genius—if genius be present where judgment is absent; not without science, if that be science which fails in precision—one of those clever desultory men who, in adopting a profession, do not give up to it the whole force and bent of their mind." Dr. Lloyd was an enthusiastic advocate of mesmerism as a curative process, and an ardent believer in the reality of somnambular clairvoyance as an invaluable gift of certain privileged organizations. Nay more, "on these beliefs he founded an argument for the existence of soul, independent of mind as of matter, and built thereon a superstructure of physiological phantasies, which, could it be substantiated, would replace every system of metaphysics on which recognised philosophy condescends to dispute." Against these doctrines Dr. Fenwick sternly opposed himself, and his vanity having been wounded by the manifestation of a little good-natured but misplaced condescension on the part of Dr. Lloyd, he put forth a small pamphlet on the subject of mesmerism, in which he "exhausted all the weapons that irony can lend to contempt." This pamphlet had, unhappily, the effect of exposing Dr. Lloyd to the ridicule of his fellow-townsmen. Popular feeling was enlisted against him, he lost his practice and repute, and mortification and anger brought on a stroke of paralysis. Time passes on; the controversy has been well nigh forgotten, when, one winter's evening, Dr. Fenwick is summoned to the bedside of his quondam opponent. He goes there to find him dying, his young and helpless children about him.

"'I have summoned you to gaze on your own work,' exclaimed the dying man. 'You have stricken down my life at the moment when it was most needed by my children, and most serviceable to mankind. Had I lived a few years longer my children would have entered on manhood, safe from the temptations of want and undejected by the charity of strangers. Through you, they will be penniless orphans. Fellow-creatures afflicted by maladies your pharmacopœia had failed to reach, came to me for relief and found it. 'The effect of imagination,' you say. What matters if I directed imagination to cure? Now you have knocked the unhappy ones out of their last chance of life. They will suffer and perish. Did you believe me in error? Still you knew that my object was research into truth. You employed against your brother in art venomous drugs and a poisoned probe. Look at me! Are you satisfied with your work? Vain pretender, do not boast that you brought a genius for epigram to the service of science. Science is lenient to all who offer experiment as the test of conjecture. You are of the stuff of which inquisitors are made. You cry that truth is profaned when your dogmas are questioned. In your shallow presumption you have meted the dominions of nature, and where your eye halts its vision, you say, 'There nature must cease;' in the bigotry which adds crime to presumption, you would stone the discoverer who in annexing new realms to her chart, unsettles your arbitrary landmarks. Verily, retribution shall await you. In those spaces which your sight has disdained to explore, you shall yourself be a lost and bewildered straggler. Hist! I see them already! The gibbering phantoms are gathering round you!'"

The wail of the youngest orphan strikes upon the ears of Dr. Fenwick as he leaves the house of death, and haunts him long afterwards. In vain he strives to throw off the impression the dying man's words, and the sad scene which accompanied them, had made upon him. It was some time before his ruffled thoughts recovered their even course; but the mind's depths had been troubled by the painful end of his rival, and unconsciously to himself a seed of doubt sown, which germinated long afterwards: and Love presently stept in to fertilize those deeper emotions which sympathy, not without a tinge of self-reproach, had transiently aroused.

In Abbot's house Dr. Lloyd had died; in the same house was born Dr. Fenwick's love for Lilian Ashleigh, "the erring, but pure-thoughted visionary," of the story. One morning in May, Dr. Fenwick, passing the gates leading to the dwelling, unoccupied from the time of Dr. Lloyd's death until then, finds them open. "The remembrance of that death-bed," he says, "came vividly before me, and the dying man's fantastic threat rang in my startled ears. An irresistible impulse"—the term popularly applied to those unresisted impulses which after events cause to be deeply fixed in the memory—" an irresistible impulse, which I could not then account for, and which I cannot account for now—an impulse the reverse of that which usually makes us turn away with quickened step from a spot that recals associations of pain—urged me on through the open gates," and in the grounds, unseen himself, he first beholds, and yields up thought and feeling to, the beautiful Lilian Ashleigh. There was much in her constitution to perplex, torment, and baffle. "It was so with her father, whom she resembled in face and character. He showed no symptoms of any grave malady: his outward form was, like Lilian's, a model of symmetry, except in this, that, like hers, it was too exquisitely delicate; but, when seemingly in the midst of perfect health, at any slight jar on the nerves he would become alarmingly ill." She recoiled from the affairs of every-day life, and having been fostered in seclusion sought her chief happiness in solitude, giving herself up unchecked to the pleasing, but dangerous, seductions of reverie. She lived chiefly in a waking dream, played upon by, and ceding without resistance to, her own emotions and the influence exercised upon her, unconsciously to herself, by others. "She is fond of reading," explains her mother, "but more fond of musing. She will sit by herself for hours without book or work, and seem as abstracted as if in a dream. She was so even in her earliest childhood. Then she would tell me what she had been conjuring up to herself. She would say that she had seen—positively seen—beautiful lands far away from earth: flowers and trees not like ours." We are introduced to her when this habit assumes a graver cast; when the illusions and hallucinations of reverie obtain such sway as to influence every-day life; when translated by the after events they are looked upon as of a quasi-prophetic character (—and the mind once possessed with the idea of their supernatural nature, is but too prone, dominated by the emotions, to work out in real life its own fantastic, uncontrolled play upon the senses); when the reverie verges upon ecstasy, and is apt to culminate in hysterical paroxysms; when the waking dream of the day is followed and continued by the acted dream—somnambulism of the night; and when even in the best moments there was a mobility of feeling and thought which marked how closely this morbid susceptibility of the system was allied to insanity—the end, the pitiable goal, to which all individuals of whom Lilian Ashleigh is the type, tend, and to which she was driven. "Her spirits were more generally light and joyous than I should have premised from her mother's previous description. She would enter mirthfully into the mirth of young companions round her; she had evidently quick perception of the sunny side of life; an infantine gratitude for kindness; an infantine joy in the trifles that amuse only those who delight in tastes pure and simple. But when talk rose into graver and more contemplative topics, her attention became earnest and absorbed, and sometimes a rich eloquence, such as I had never before nor since heard from lips so young, would startle me into wondering silence, and even into disapproving alarm. For the thoughts she then uttered seemed to me too fantastic, too visionary, too much akin to the vagueness of a wild, though beautiful, imagination."

We may not do more than touch upon one or two points in the account put into Lilian's own mouth of the hallucinations of her reveries, after she has yielded up her young heart to Dr. Fenwick. She tells how at times she saw the face of her lost father, and heard his very voice as she had heard and seen him in early childhood; how for weeks before her mother had taken up her abode in the Abbot's house, and before she knew that such a place existed, she had seen distinctly the old house, the surrounding trees, the sward, the moss-grown Gothic fount; and with the sight the impression had been conveyed to her that in the scene her then childlike age would pass into solemn change: and how on the evening when Dr. Fenwick first saw her gazing towards the Orient, she saw him in her vision, and her father, who whispered, "Ye will need one another;" and the head of a third—a young head—changing like a Medusa's, and threatening and boding mischief.

Two ghosts are next intruded in the story. Our lovers are separated for a short time, and to rouse himself from the "aching sense of void and loss," Dr. Fenwick earnestly resumes an ambitious work on "Organic Life," in which he had been engaged two years, but which had been laid aside during the agitating period of his early courtship. The very night of the day on which Lilian had left home he reopened his MS.; and while complacently and warmly engaged in meting out limits to natural laws and constructing Intellectual Man out of his material senses (heedless even of the strange and deeper mysteries of feeling which his love for Lilian might have taught him), he is startled by a sigh:—"Suddenly beside me I heard a sigh—a compassionate, mournful sigh. The sound was unmistakeable. I started from my seat, looked round, amazed to discover no one—no living things! The windows were closed, the night still. That sigh was not a wail of the wind. But, there, in the darker angle of the room, what was that? A silvery whiteness—vaguely shaped as a human form—receding, fading, gone! Why, I know not, for no face was visible, no form, if form it were, more distinct than the colourless outline;—why, I know not, but I cried aloud, 'Lilian! Lilian!'"—He smiled and blushed at his folly. Here was an anecdote at his own expense, which might well be quoted in his Chapter on the Cheats of the Senses and Spectral Phantasms. He writes on until his lamp wanes in the grey of the dawn. His pride triumphs in the thought, as he lays down to rest, that he has incontrovertibly established his creed of materialism, and perhaps written that which would found a school and form disciples, when he again hears the sigh. But this time it caused no surprise, and murmuring, "Certainly a strange thing is the nervous system!" he turns on his pillow, and falls asleep.

The next day (so closely does one marvel tread upon the heels of another) Dr. Fenwick is summoned in haste to attend the steward of a Sir Philip Derval, not residing at his family seat, which was about five miles from L——. This man, somewhat advanced in years, but of robust conformation, had a few hours before been seized with a fit, supposed to be apoplectic, but had recovered so far as to have become sensible and out of immediate danger. The fit had been ushered in by a spectral apparition of his master, then abroad. This story, the doctor concludes, seemed at once to decide the nature of the fit to be epileptic; but he afterwards adds, "Riding homewards, I mused on the difference that education makes, even pathologically, between man and man. Here was a brawny inhabitant of rural fields, leading the healthiest of lives, not conscious of the faculty we call imagination, stricken down almost to Death's door by his fright at an illusion,"—explicable on the same grounds as those which had given rise to the spectre which had tricked the doctor's imagination the night before, and which he had treated with such sublime indifference, thanks to his more gifted mind. Is it not more consistent with fact and observation to argue, when an optical illusion or hallucination precedes an apoplectic or epileptic fit, that both arise from the same morbid condition, and not that the latter is the result of the emotion excited by the former?

A pleasant evening gossip now brings on the scene Sir Philip Derval, and paves the way for the introduction of the third great character in the romance—Margrave, "the image of sensuous, soulless Nature." Contemporaneously with the appearance of these gentlemen in the story, and by their agency, we are led into a higher region of the Marvellous, for reception into which the mind, it is to be presumed, has been fittingly prepared by the more commonplace marvels which have, with no niggardly hand, been dealt out to the reader.

(*To be continued.*)

